# Examining Passively Collected Smartphone-Based Data in the Days Prior to Psychiatric Hospitalization for a Suicidal Crisis: Comparative Case Analysis

**DOI:** 10.2196/55999

**Published:** 2024-03-20

**Authors:** Ross Jacobucci, Brooke Ammerman, Nilam Ram

**Affiliations:** 1 Department of Psychology University of Notre Dame Notre Dame, IN United States; 2 Departments of Communication and Psychology Stanford University Stanford, CA United States

**Keywords:** screenomics, digital phenotyping, passive assessment, intensive time sampling, suicide risk, suicidal behaviors, risk detection, Comparative Analysis, suicide, suicidal, risk, risks, behavior, behaviors, detection, prediction, Smartphone-Based, screenomic, case review, participant, participants, smartphone, smartphones, suicidal ideation

## Abstract

**Background:**

Digital phenotyping has seen a broad increase in application across clinical research; however, little research has implemented passive assessment approaches for suicide risk detection. There is a significant potential for a novel form of digital phenotyping, termed screenomics, which captures smartphone activity via screenshots.

**Objective:**

This paper focuses on a comprehensive case review of 2 participants who reported past 1-month active suicidal ideation, detailing their passive (ie, obtained via screenomics screenshot capture) and active (ie, obtained via ecological momentary assessment [EMA]) risk profiles that culminated in suicidal crises and subsequent psychiatric hospitalizations. Through this analysis, we shed light on the timescale of risk processes as they unfold before hospitalization, as well as introduce the novel application of screenomics within the field of suicide research.

**Methods:**

To underscore the potential benefits of screenomics in comprehending suicide risk, the analysis concentrates on a specific type of data gleaned from screenshots—text—captured prior to hospitalization, alongside self-reported EMA responses. Following a comprehensive baseline assessment, participants completed an intensive time sampling period. During this period, screenshots were collected every 5 seconds while one’s phone was in use for 35 days, and EMA data were collected 6 times a day for 28 days. In our analysis, we focus on the following: suicide-related content (obtained via screenshots and EMA), risk factors theoretically and empirically relevant to suicide risk (obtained via screenshots and EMA), and social content (obtained via screenshots).

**Results:**

Our analysis revealed several key findings. First, there was a notable decrease in EMA compliance during suicidal crises, with both participants completing fewer EMAs in the days prior to hospitalization. This contrasted with an overall increase in phone usage leading up to hospitalization, which was particularly marked by heightened social use. Screenomics also captured prominent precipitating factors in each instance of suicidal crisis that were not well detected via self-report, specifically physical pain and loneliness.

**Conclusions:**

Our preliminary findings underscore the potential of passively collected data in understanding and predicting suicidal crises. The vast number of screenshots from each participant offers a granular look into their daily digital interactions, shedding light on novel risks not captured via self-report alone. When combined with EMA assessments, screenomics provides a more comprehensive view of an individual’s psychological processes in the time leading up to a suicidal crisis.

## Introduction

### Background

In 2021, a total of 48,183 Americans died by suicide, with rates rising in the United States up to 2019 [1] and early indications of a continuing trend into 2022 [2]. While the number of suicides continues to increase, our ability to predict suicidal thoughts and behaviors (STBs) remains stagnant [3], underscoring the potential benefits of shifting focus from identifying who is at risk to when individuals are at risk for suicide. Studies using ecological momentary assessment (EMA) to collect data multiple times per day have demonstrated that key suicide risk factors, including suicidal ideation, change rapidly across the course of the day [4]. However, this research has been limited by the temporal granularity of assessments (ie, every 3-4 hours). It has yet to pay due attention to prospectively predicting suicidal behaviors (ie, suicidal planning and suicide attempts) [5]. Notably, the transition from contemplating suicide to deciding to act on such thoughts can transpire in as little as 10 minutes [6,7], making this decision-making process an ideal candidate for study in temporally intensive designs.

Intensive time sampling studies that use EMA have been shown to capture suicide risk processes effectively [8]; however, these methodologies carry a high participant burden, with the expectation that participants are willing and able to actively report on their experiences consistently throughout and across several days, including during times of distress. This contributes to the high rates of missing data within EMA designs, with compliance rates often around 60% in clinical populations [8]. Despite these limitations, asking self-report items at an intensive time scale has shed light on the dynamic nature of STB [4], with recent work becoming increasingly granular, prompting assessments every 10 minutes for an hour [9]. Furthermore, EMA research has also shed more light on the dynamic influence (both concurrent and prospective) of theoretically identified constructs [10], with a prominent focus on theoretically relevant risk factors, such as thwarted belongingness and perceived burdensomeness [11].

In contrast to active assessment approaches, the reliable detection of experiences within a relatively short window (eg, minutes) can be enhanced by technologically innovative methodologies that can passively and continuously capture the dynamic nature of factors contributing to suicide risk. This has provided, in part, the foundation for the surge in applications of passive sensing—including digital phenotyping [12]—in clinical research. Digital phenotyping is the use of data from smartphones and other personal digital devices to measure behaviors, cognitive functions, mood, and other psychological variables [13,14]. By collecting and analyzing data such as keystrokes, voice patterns, geolocation, social media activity, and other digital traces, digital phenotyping offers a real-time, high-resolution, yet low burden, approach to understanding and monitoring clinical phenomena.

Digital phenotyping has seen an increase in application across clinical research broadly, with the term being previously applied to suicide risk profiles based on active assessments (ie, EMA) [15]. However, little research has specifically targeted passive assessment approaches to suicide risk detection, with existing findings being mixed concerning its predictive validity. For example, in a recent study, Czyz et al [16] found that sensor-based assessments (resting heart rate, heart rate variability, step count, and sleep duration) did not add incremental validity to the prediction of near-term suicidal thoughts above and beyond EMA self-report questions among high-risk adolescents. Alternatively, it was demonstrated that psychological monitoring of electrodermal activity did improve fit upon EMA-only models in the prediction of suicidal ideation severity [17]. The limited work in this realm leaves open the possible use of passive assessment in better understanding suicide risk.

One of the most recently available methods in performing digital phenotyping is screenomics [18,19], wherein participants passively provide temporally intensive records of their psychological and social life as it manifests on the screens of their digital devices [19]. In this case, screenomes, or the record of experiences on digital devices with screens [19], are captured via software that takes screenshots of an individual’s smartphone screens every 5 seconds when the device is in use. Initial work using screenomics has described the timing and patterns of smartphone usage by extracting various textual and graphical features from screenomes, allowing for a mapping of app use and topic of content viewed [20], among other key use features such as the emotion valence of content viewed [21]. More recently, screenomes have informed the examination of digital communications, including differentiation between produced (ie, actively producing smartphone content) and consumed (ie, passive consumption) communication content [22], as well as the communication dynamics within relationships [23].

### This Study

Following the possibility of using screenomes to learn about individuals’ emotional experiences and interpersonal dynamics, this study is the first, to our knowledge, to engage screenomics with a clinical population and, more specifically, for the study of suicide risk. This paper focuses on a comprehensive case review of 2 participants, detailing their passive (ie, obtained via screenomics screenshot capture) and active (ie, obtained via EMA) risk profiles that culminated in suicidal crises and subsequent psychiatric hospitalization. In our approach, we intentionally focused on building a qualitative, rather than quantitative, understanding of the data, purposely eschewing statistical modeling to capture the nuanced individual experiences and complexities inherent in suicide risk. Further, we iteratively followed inductive and deductive approaches [24,25] in deciding which variables to define (in passively collected data) and focus on (in actively collected data). Through this analysis, we shed light on the timescale of risk processes as they unfold prior to hospitalization, as well as introduce the novel application of screenomics within the field of suicide research. To underscore the potential benefits of this method in comprehending suicide risk, we concentrate on a specific type of data gleaned from screenshots—text—captured prior to hospitalization, alongside self-reported EMA responses.

## Methods

### Overview

**Figure 1:**
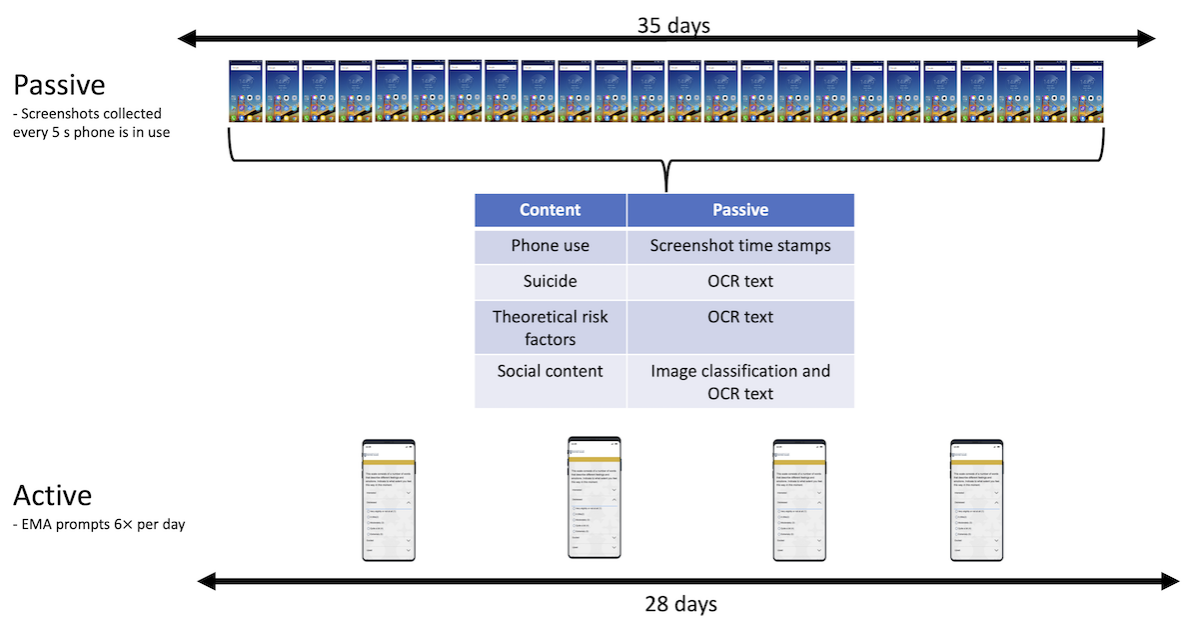
General overview of the study design. EMA: ecological momentary assessment; OCR: optical character recognition.

This case review focuses on 2 participants who had suicidal crises and subsequent psychiatric inpatient treatment, coincident with their participation in an intensive time-sampling study that included both active and passive assessment approaches. To participate in the larger study, participants were required to have an Android smartphone, daily access to the internet (via Wi-Fi or cellular data), a past 1-month history of active suicidal ideation, and no history of being diagnosed with schizophrenia. Participants completed a baseline assessment, 28 days of active assessments obtained via signal-contingent EMA prompts, and 35 days of passive assessments obtained via screenshot collection. Participants were randomly assigned to 2 protocols where they provided the additional 7 days of passive screenshot collection (with no EMA prompts) in the week prior to the 28-day EMA+screenshot collection period (person A) or the week following the 28-day EMA+screenshot collection period (person B). For the 2 participants included in this study, data collection occurred before, during, and after an acute crisis and hospitalization spanning 38 (person A) and 51 (person B) days, respectively. A general overview of the data collected and how the screenshots were processed is depicted in Figure 1.

### Ethical Considerations

All procedures were approved by the institutional review board of the University of Notre Dame (protocol number 21-12-6965). Prior to engaging in any study procedures, participants were provided a full description of the study and the opportunity to ask questions, including speaking with study principal investigators (BA and RJ) or the institutional review board if any concerns were expressed. All participants provided written informed consent. They were compensated up to US $215 for participation (ie, US $40 for baseline assessment, US $100 for a 28-day EMA period, US $20 for completing at least 75% of EMA surveys, and US $55 for a 35-day screenomics period). Self-reported data from this study are deidentified; screenshot data are coded with layered IDs, encrypted upon storage upload, processed to remove potentially identifying information (ie, names and addresses), and accessible only to core study team members.

### Active Assessment

#### Overview

Across the 28 days, participants received 6 prompts each day to complete a short survey on their smartphone (ie, signal-contingent design); prompts were delivered randomly within 2-hour windows across a 12-hour participant-selected block (eg, 9 AM to 9 PM). When participants received a prompt, they had 30 minutes (with one 15-minute reminder) to complete the short survey (expected to take <3-4 minutes). The EMA protocol was administered via the LifeData software on participants’ smartphones. To improve compliance and data quality, participants completed a “practice” EMA signal-contingent prompt during the baseline session and were incentivized for completing ≥75% of EMA signal-contingent responses (determined every week); EMA compliance was calculated based on the number of prompts received versus the number of prompts completed. We identified “high” scores for each variable depicted below by calculating whether an individual score was at or above the 90th percentile based on the person-specific empirical distribution.

#### Risk Factors

For this paper, we primarily focus on 2 key risk factors related to suicide: thwarted belongingness and perceived burdensomeness, as explained in the Interpersonal Psychological Theory of Suicide [26]. However, we also examined other important risk factors supported by prior research, including positive and negative emotions, urges, and behaviors related to nonsuicidal self-injury, alcohol and drug use urges, interpersonal conflict, self-reported sleep patterns, and physical pain [10]. The majority of these items were answered on a 5-point Likert scale, except interpersonal conflict, self-injurious and alcohol use behaviors, and nightmares, which were binary. Further, assessments of pain were reported on a 0-100 scale. Multimedia Appendix 1 [27,28] shows details for EMA questions to access risk factors.

#### Suicide Risk

##### Suicidal Ideation

Four questions assessed momentary (ie, “In this moment…”) passive (ie, “Life is not worth living for me”; “There are more reasons to die than to live”) and active (ie, “I want to die”; “I think about taking my life”) suicidal ideation, all items were answered on a 5-point Likert scale. Based on prior work by the study team, which showed that the 4 items form 1 factor (rather than 2 factors) [11], the item responses were summed to obtain a composite momentary suicidal ideation score.

##### Suicidal Planning

Three items assessed suicidal planning since the last prompt (ie, “Considered a specific suicide method”; “Identified how to acquire your suicide method”; “Made other preparations for your death”); these items were summed to create a composite suicidal planning score. All items were answered on a 5-point Likert scale.

##### Suicidal Desire

Two questions assessed momentary suicidal urges (ie, “How strong is your urge to make a suicide attempt?”; “How intense is your desire to kill yourself?”); items were answered on a 5-point Likert scale. These items were summed to create a composite suicidal desire score.

### Passive Assessment

#### Overview

Continuous smartphone data collection was collected via the ScreenLife Capture [29] app, which is tailored for integration with HIPAA (Health Insurance Portability and Accountability Act of 1996)-compliant servers at the University of Notre Dame. This software passively captures screenshots of participants’ Android smartphone screens at 5-s intervals during smartphone use and stores the screenshots locally on the smartphone before bundling, encrypting, and transmitting them to research servers at intervals that accommodate bandwidth and device memory constraints. The screenshots are obtained continuously as the participant uses their smartphone in everyday life without requiring active engagement from the participant. In postprocessing, the screenshots’ content features are extracted using various image processing tools. In postprocessing, the text from each screenshot was extracted using EasyOCR, an open-source optical character recognition tool for Python, known for its efficiency in processing images containing text, even under less than perfect clarity or with intricate layouts. Optical character recognition technology converts various document types, such as scanned paper documents, PDF files, or images taken by a digital camera, into editable and searchable data. We removed any text extracted with a confidence value of less than 0.70. The text was then normalized by lemmatization, case reduction, and removal of punctuation and numbers and examined using a variety of dictionaries.

#### Risk Factor–Related Content

##### Linguistic Inquiry and Word Count

The first dictionary-based method that we tested is the linguistic inquiry and word count (LIWC) [30], which is implemented as a commercially available software program [31]. LIWC analyzes text according to a dictionary comprised of 6400 words, word stems, and emoticons, primarily related to psychological processes (eg, affective and social processes) [31]. The output contains scores on 90 dictionary components and linguistic or grammar dimensions of their data. For this paper, we only extracted components that we thought could be related to psychological processes or risk factors for suicide (eg, substances). Multimedia Appendix 2 [31] shows an overview of LIWC components. To display the scores, we opted to depict a ratio (score for each day/total) score for each instead of the absolute number of mentions for each category.

##### Topic-Specific Dictionaries

Since the specific words in the LIWC components are proprietary, we also created our own dictionary of common words likely associated with these components, particularly those crucial for understanding the suicide risk process. For example, we created a substances dictionary. Multimedia Appendix 3 contains words used in specific dictionaries.

##### Social Content

We identified which smartphone app was in use through the text portrayed when an app was in use. After examining participants’ screens to identify phone-specific app displays, we used the following words to identify text messaging (SMS): “text message,” “sms,” and “text,” while the following text for Meta: “write a comment,” “what’s on your mind,” “like,” “facebook,” and “Instagram.” We acknowledge that this dictionary will not perfectly distinguish between each app and similar apps, with a more robust method needed when extrapolating beyond these 2 individuals. Additionally, we used external data to train a model to identify whether the screenshot contained indicators of using a social app. We used the Rico data set [32], a repository of 66,261 screen images from Android operating systems. Each screenshot in Rico captures a momentary snapshot of a mobile app’s user interface. For our purposes, one of the crowdsourced annotations is “Is_Social” (4172 positive cases), which classifies whether or not a screen contains social media elements or functionalities. From this, we randomly split the images into an 80/20 train-test split and fine-tuned ResNet18, which is a variant of the Residual Network family, a type of convolutional neural network architecture. The model was trained for 50 epochs, choosing a final model based on the test set area under the curve score, which was maximized at 0.89. We applied this model to each person’s screenshots, aggregating the predicted probabilities by day.

##### Suicide Language

We defined our dictionary to derive scores specific to suicide-related words (ie, “kill myself” and “end it all”) and a separate dictionary based on theoretically linked risk factors (ie, “trapped” and “crying”). These dictionaries were created through literature review and expert consultation. Multimedia Appendix 3 contains words used in specific dictionaries. Finally, we used the dictionary, termed crisis, which was developed by screening crisis chat messages [33]; this dictionary contains 227 words, ranging from specific states regarding suicidality (ie, “hurt myself” and “I just want this all to end”), suicide-related emotion states (ie, “hate my life” and “hopeless”), and potential suicidal behavior–related words (ie, “drown” and “rope”). To clean the text, we first removed screens that contained EMA prompts, namely, to avoid capturing suicide words that were used as a part of the EMA assessments.

## Results

### Case Overview

#### Person: A

##### Overview

Person A was a 37-year-old, straight, White, non-Hispanic individual born female and identifies as a woman. They reported being single, employed full-time, and with an annual household income of US $30,000-US $39,000. This participant’s diagnostic assessment revealed they met the diagnostic criteria for current major depressive disorder, panic disorder, agoraphobia, generalized anxiety disorder, posttraumatic stress disorder, and cannabis use disorder. They reported having engaged in prior individual counseling, as well as spiritual counseling, and that they were currently taking medication for psychiatric and medical reasons, including heart problems and epilepsy. Concerning suicide risk history, they reported chronic suicidal ideation, beginning at age 10 years and attempting suicide on 5 occasions, with the first attempt at age 11 years and the most recent 2 attempts in the year before study enrollment (1 in a month prior). In the week prior to enrolling in the study, they reported thinking about suicide 14 times and denied any suicidal planning.

##### Provision of Active and Passive Data

Person A responded to EMA prompts on 25 days; on these days, they, on average, only completed 4.04 prompts (SD 1.70), resulting in compliance of 60.1% (101 completed prompts out of 168 total prompts) or 67.3% counting only the days in which they responded at least once. They provided 139,276 screenshots. An average day involved 5.92 (SD 2.75) hours of screenshot data collection.

##### Comprehensive Suicide Risk Assessments

To ensure participant safety, EMA responses that indicated elevations in imminent suicide risk triggered comprehensive risk assessments by study staff. Information obtained during these calls is not presented among active assessment results but rather provided in the Results section to provide a more in-depth contextual understanding of each detailed participant. During study enrollment, person A’s responses resulted in a comprehensive suicide risk assessment 9 times. Through these calls, person A expressed feelings of being overwhelmed and hopeless; they also expressed difficulties with having limited social support. They engaged in means restriction pertaining to their most recent suicide attempt method. Suicidal ideation was reported on these calls; however, the participant denied suicidal planning or intent. Day 26 in the study was the last time person A’s responses triggered a suicide risk assessment (study staff unable to reach them after 3 attempts) prior to their choice for hospitalization; however, on day 29 of participation, the participant reported self-admitting for psychiatric hospitalization due to concerns of being unable to keep themself safe in the context of their suicidal thinking, and study staff spoke with them after they were on the way to the hospital due to EMA responses triggering a suicide risk assessment. They were hospitalized on days 29-32 of study participation.

#### Person B

##### Overview

Person B was a 29-year-old, White, non-Hispanic individual who was born female and has a nonbinary gender identity; they reported questioning their sexual identity at the time of baseline assessment. They reported being employed full-time, living with a partner, and having an annual household income of US $40,000-US $49,000. This participant’s diagnostic assessment revealed they met current diagnostic criteria for bipolar I, as well as current panic disorder, social anxiety disorder, and substance use disorders (cannabis, amphetamine, and cocaine). They reported having previously engaged in several forms of outpatient treatment (ie, individual counseling, group counseling, couples counseling, and day treatment) and currently taking medication for medical and psychiatric reasons. Stated medical concerns included heart and respiratory problems.

Concerning suicide risk history, person B reported chronic suicidal ideation, experiencing suicidal thoughts on 95% of days, beginning at the age of 7 years. They also reported having attempted suicide on 4 occasions, with the first being at the age of 13 years, and the most recent 5 years prior to study involvement. In the week prior to enrolling in the study, they reported thinking about suicide 7 times and planning for suicide 3 times.

##### Provision of Active and Passive Data

Person B completed EMA surveys on 23 days, responding to, on average, 2.7 (out of 6; SD 1.63) prompts per day, resulting in a compliance of 37.5% (63 completed prompts out of 168 total prompts) or 45.6% counting only the days in which they responded at least once. Participant B provided 58,098 screenshots. An average day involved 3.59 (SD 2.74) hours of screenshot data collection.

##### Comprehensive Suicide Risk Assessments

During study enrollment, person B’s responses to the suicide-specific EMA questions triggered the study risk assessment protocol 8 times. During these calls, the participant expressed their suicidality was prompted by a panic attack, as well as interpersonal conflict that was impacting their housing stability. Suicidal ideation and suicidal planning were expressed in multiple calls; however, intent to act on these plans was low, and confidence in keeping themselves safe was high. They reported the use of several coping strategies, such as connecting with their care provider (resulting in adjustments to their psychiatric medication), means restriction, reviewing reasons for living, future planning, listening to music, sitting with their emotions, and deep breathing. However, on day 18 of participation, the participant attempted suicide and subsequently received inpatient psychiatric treatment from days 18 to 22; person B emailed the study staff to notify them of hospitalization after discharge (ie, day 22).

### Comparative Analysis

In the following sections, we describe each person’s active assessment and passive assessment risk profiles surrounding their suicidal crises and subsequent hospital admission. The vertical red line in the visualizations represents the first day of each person’s hospitalization.

#### General Use Patterns

##### Active Assessment Compliance

**Figure 2:**
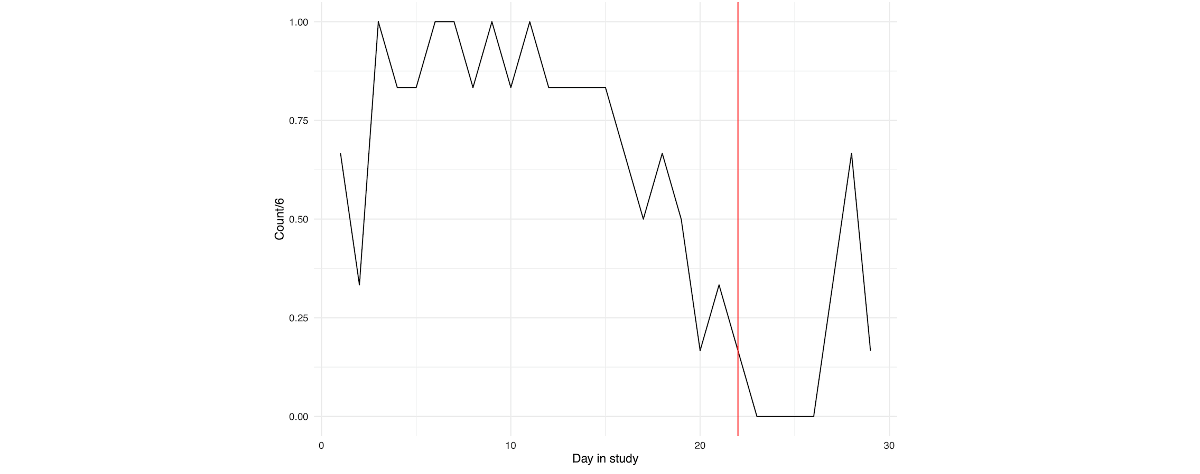
Person A: ecological momentary assessment compliance across the study. The vertical red line denotes the first day of hospitalization.

**Figure 3:**
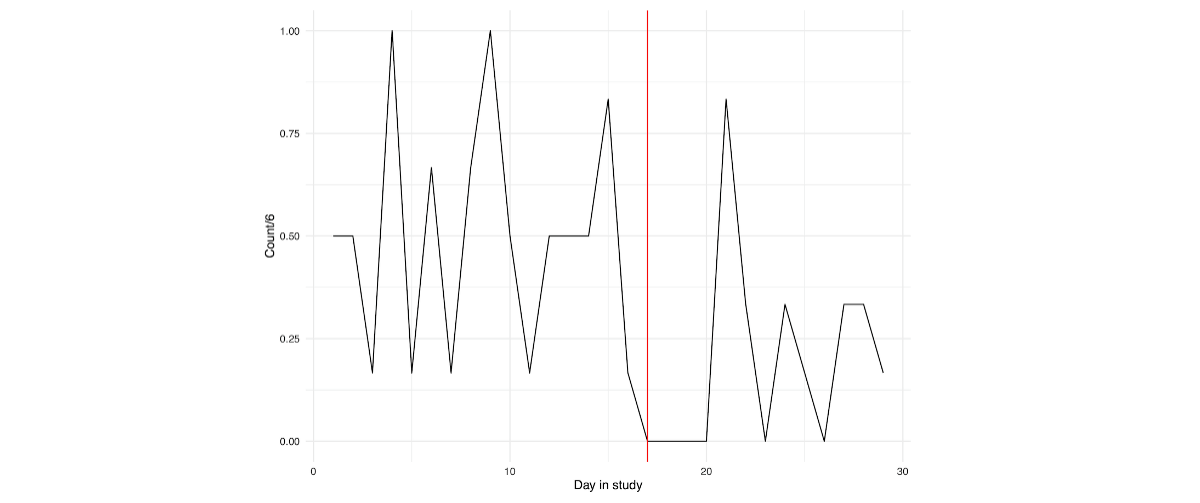
Person B: ecological momentary assessment compliance across the study. The vertical red line denotes the first day of hospitalization.

For both individuals, we can see that EMA compliance was lowest surrounding their hospitalization. Particularly for person A (Figure 2), we see a decline in compliance with the active assessment protocol leading up to hospitalization, with a more precipitous decline for person B (Figure 3). For person A, the nonprovision of active assessments presents several problems for assessing suicide risk via EMA, as it suggests that some individuals may be less likely to respond to research survey prompts during periods when they are at the highest risk.

##### Phone Use

**Figure 4:**
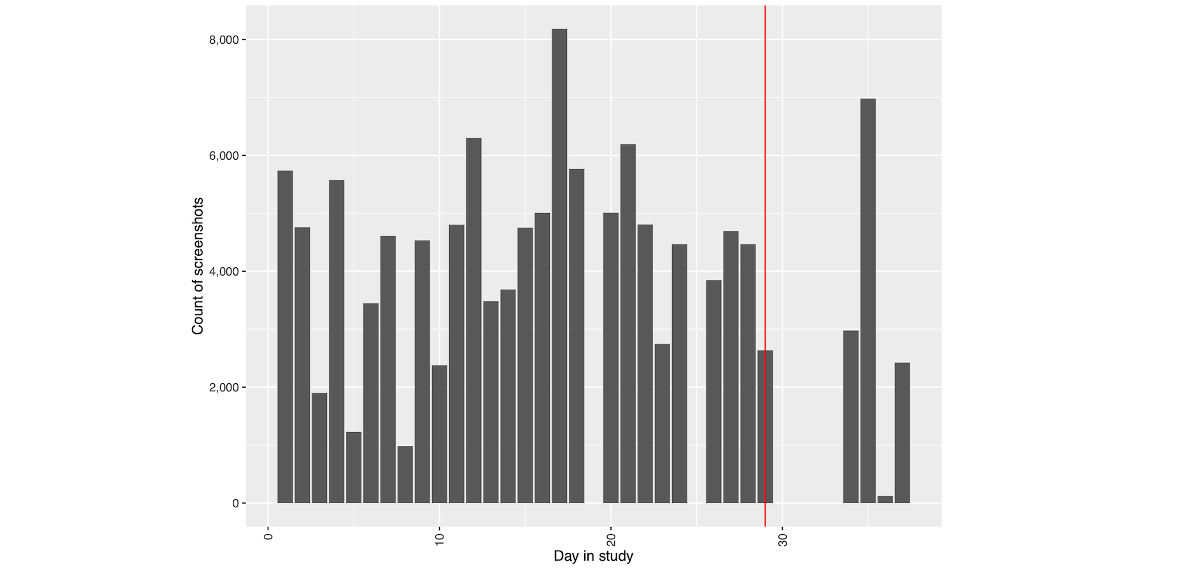
Person A: number of screenshots by day in study. The vertical red line denotes the first day of hospitalization.

**Figure 5:**
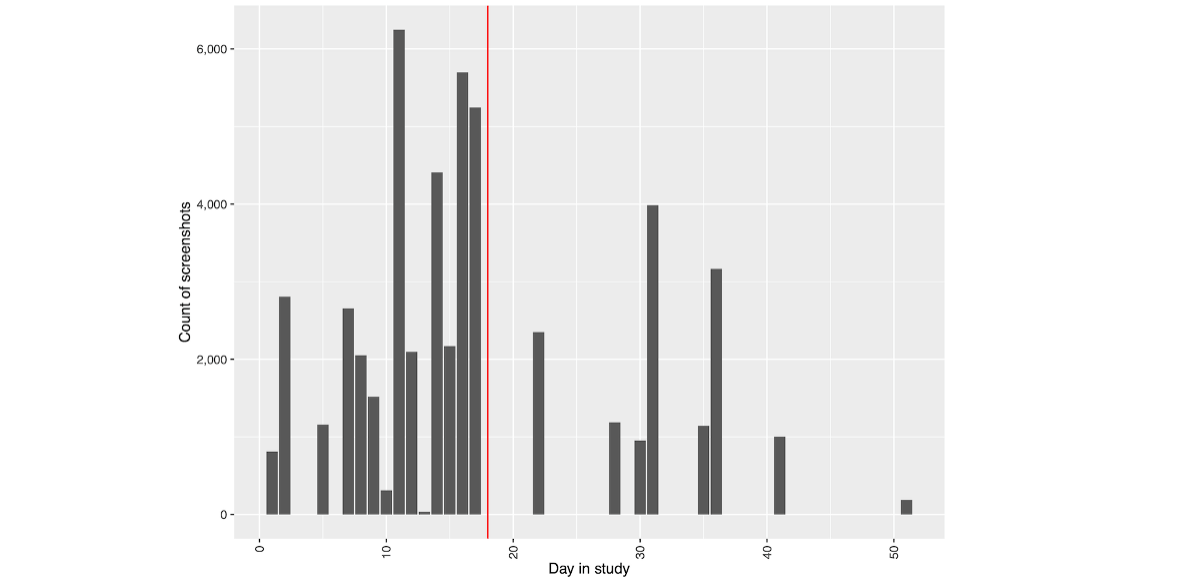
Person B: number of screenshots by day in study. The vertical red line denotes the first day of hospitalization.

We see a different picture regarding phone use leading up to hospitalization, as depicted in Figures 4 and 5. While the number of passively collected screenshots stays relatively consistent leading up to the hospitalization for person A (Figure 4), we see an increase in phone use for person B (Figure 5) the day prior.

**Figure 6:**
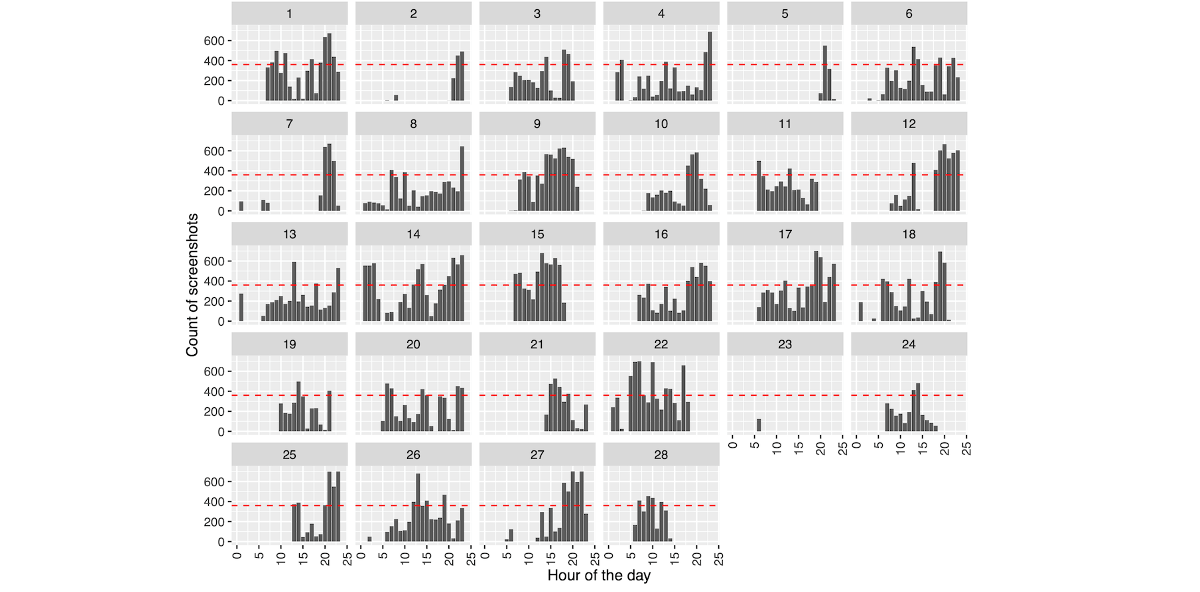
Person A: screenshot count across days and hours of each day. The horizontal red line depicts 50% phone use for that hour.

**Figure 7:**
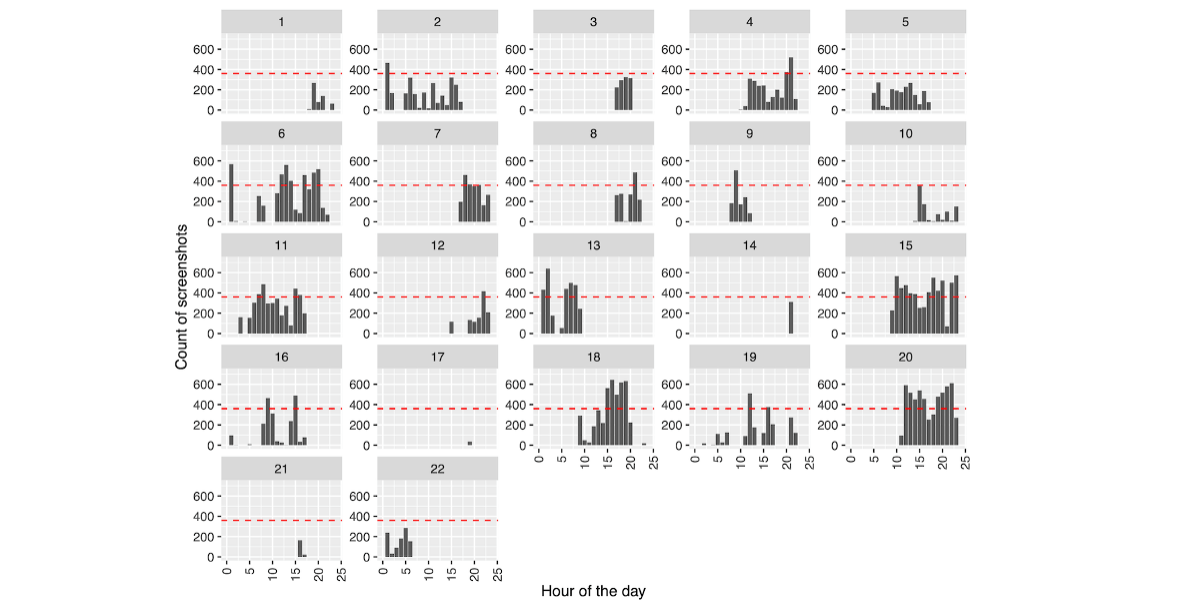
Person B: screenshot count across days and hours of each day. The horizontal red line depicts 50% phone use for that hour.

**Figure 8:**
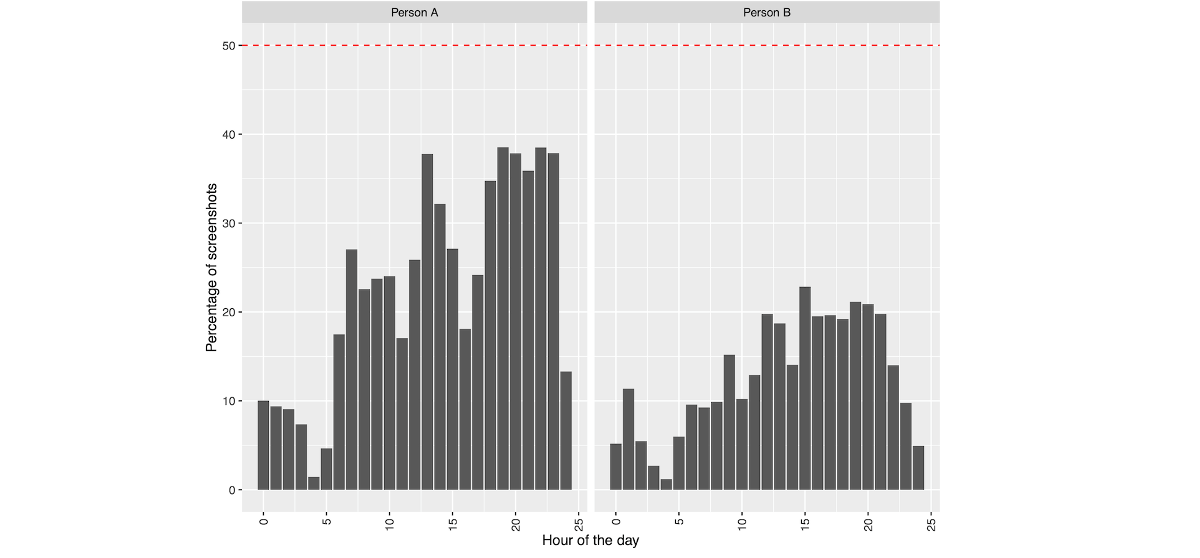
The average percentage of each hour that screenshots were collected. Person A is in the left panel and person B is in the right. The horizontal red line denotes 50% use by hour.

Phone use leading up to hospitalization can be further decomposed to look at the timing of when screenshots were collected within a given day; see Figures 6 and 7, where a horizontal red line denotes 50% phone use (ie, 30 minutes=360 five-second intervals) for that hour. For person A (Figure 6), we can see relatively consistent phone use during specific times of the day, along with periods in which their phone is not in use. Most days include at least 1 hour in which they were intensively using their phone (>50% time spent on the phone). In contrast, person B (Figure 7) was more variable in their phone use—some days included periods of intense use, while on others, phone use was sparse. Notably, neither person A nor B’s screenshot patterns indicate a consistent use pattern. The above phone use patterns do not suggest poor sleeping habits as revealed by periods of nonuse; however, we further examine this by considering phone use across hours of the day, aggregated across days. We calculated the percentage of each hour, on average, that each person used their phone (Figure 8: the horizontal red line represents 50% [ie, 30 minutes] average use for that hour). On average, neither person approaches using their phone 50% of the time. As mentioned earlier, person B (Figure 8: right) uses their phone less than person A (Figure 8: left). However, we see similar trends across individuals, with increasing phone use across the day. Notably, we also see some degree of either variable sleep schedules or sleep schedules being interrupted by phone use, as there are few hours of the day with no phone use for either person.

#### Active Assessment

##### Risk Factors

**Figure 9:**
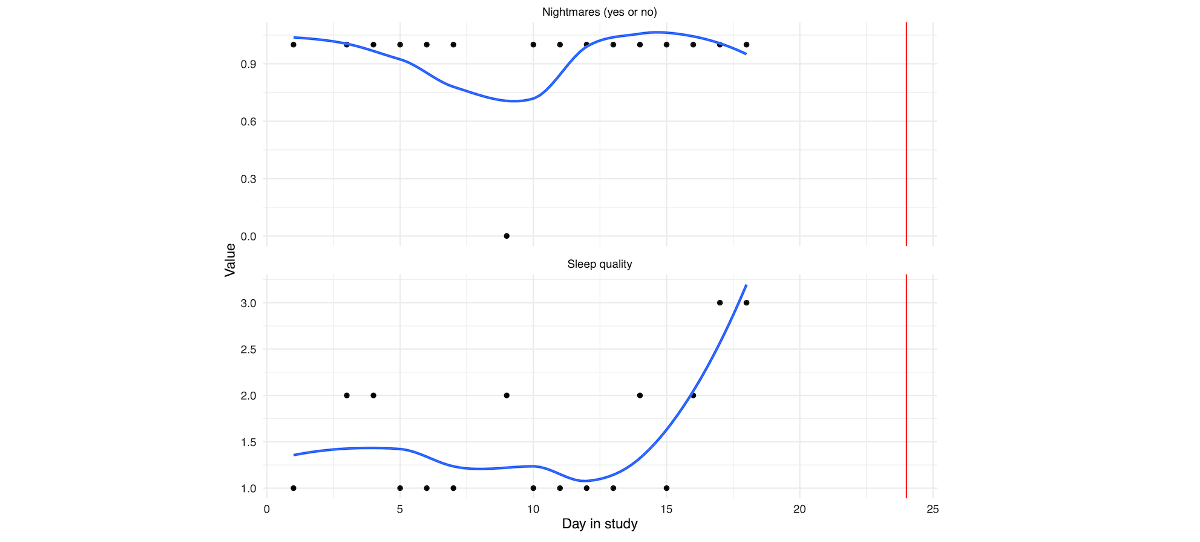
Person A: ecological momentary assessment sleep responses across days in the study. Nightmares (yes or no) in the top panel, subjective sleep quality in the bottom panel. The vertical red line denotes the first day of hospitalization.

**Figure 10:**
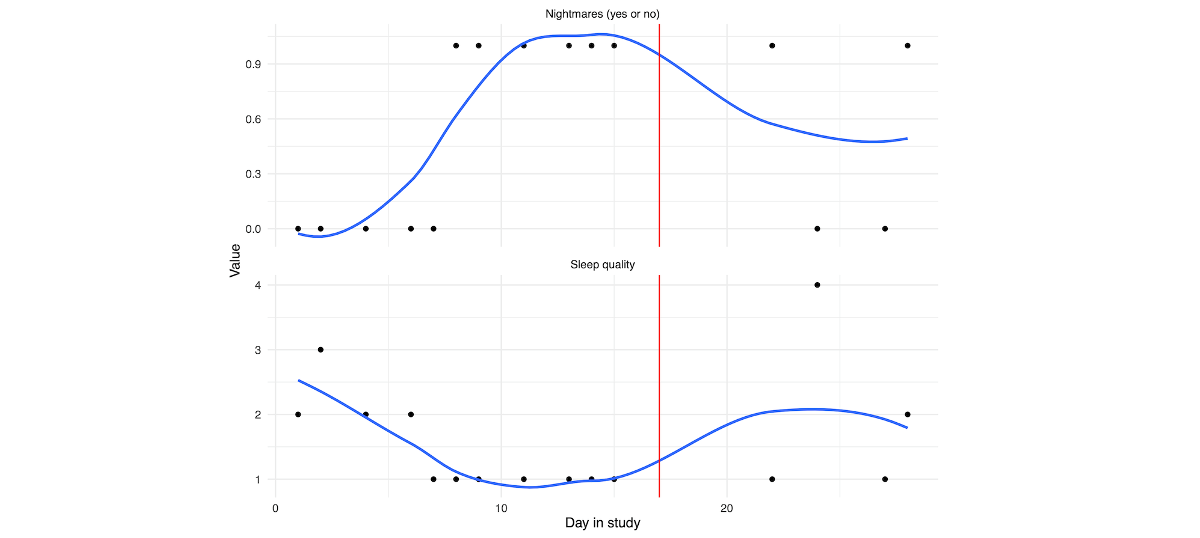
Person B: ecological momentary assessment sleep responses across days in the study. Nightmares (yes or no) in the top panel, subjective sleep quality in the bottom panel. The vertical red line denotes the first day of hospitalization.

Self-reported sleep indicators were assessed each morning. Subjective quality of sleep (sleep quality: bottom panel) and the presence of nightmares (top panel: nightmares yes or no) are depicted in Figure 6 (person A: Figure 9 and person B: Figure 10).

Compliance was particularly low for this set of questions. However, there are clear trends for both. While person A had consistent nightmares leading up to the hospitalization, their sleep quality improved (5=very good night of sleep). This is in contrast to person B, where nightmares increased and sleep quality decreased prior to the hospitalization.

**Figure 11:**
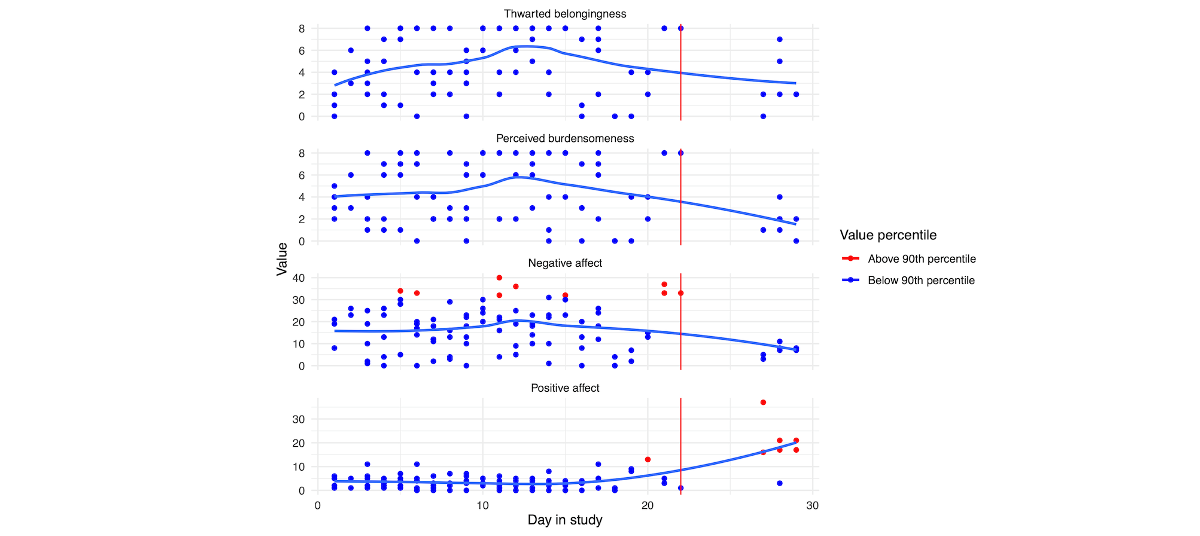
Person A: risk factor ecological momentary assessment responses across days in the study. The blue line is a loess curve, while the vertical red line denotes the first day of hospitalization.

**Figure 12:**
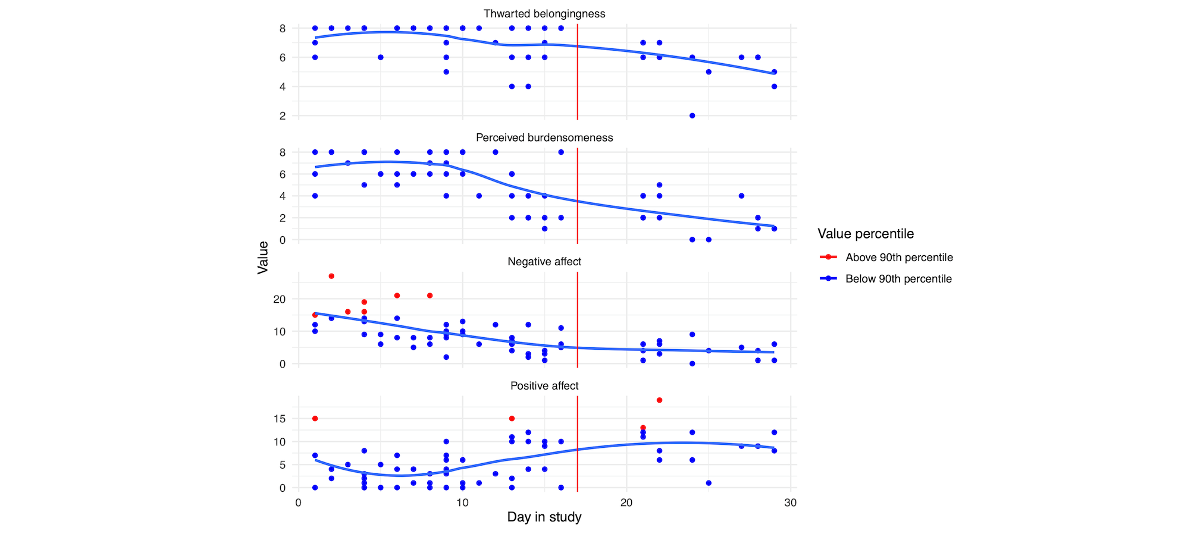
Person B: risk factor ecological momentary assessment responses across days in the study. The blue line is a loess curve, while the vertical red line denotes the first day of hospitalization.

Of theoretically and empirically relevant variables that we assessed via EMA, we do not see any increasing trends in perceived burdensomeness, thwarted belongingness, negative affect, or positive affect in Figures 11 and 12 (notably, however, there are a few spikes in negative affect for person A immediately prior to hospitalization). Figures 11 and 12 depict the empirically supported risk factors that demonstrated change leading up to hospitalization. We can see that both individuals reported consistent interpersonal conflicts across the study. However, there were no clear increases leading up to the hospitalization. For person A (Figure 11), we do see a slight increase in urges to drink alcohol; however, when we look at the responses related to alcohol use, they only reported drinking alcohol on 2 days. Further, both individuals report consistent urges to use drugs, with person B exhibiting a slight increase in urges prior to hospitalization. Finally, person A reported moderate urges to self-injury, with reported self-injury engagement on 1 occasion. However, there is no clear pattern of changes in risk factors prior to hospitalization.

##### Suicide Risk

**Figure 13:**
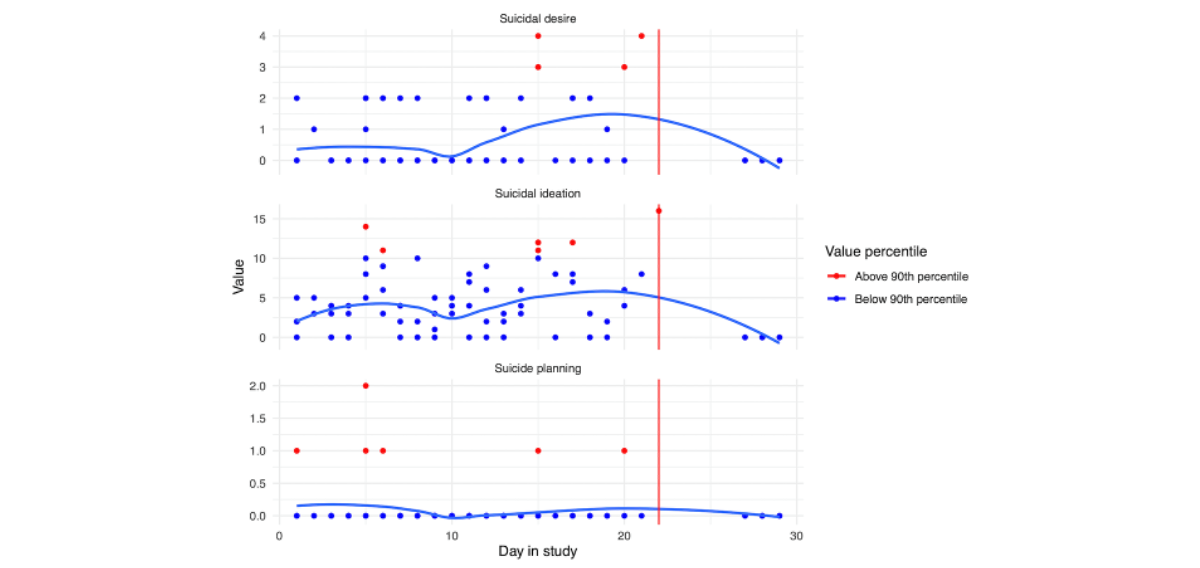
Person A: suicide ecological momentary assessment responses across days in the study. The blue line is a loess curve, while the vertical red line denotes the first day of hospitalization.

**Figure 14:**
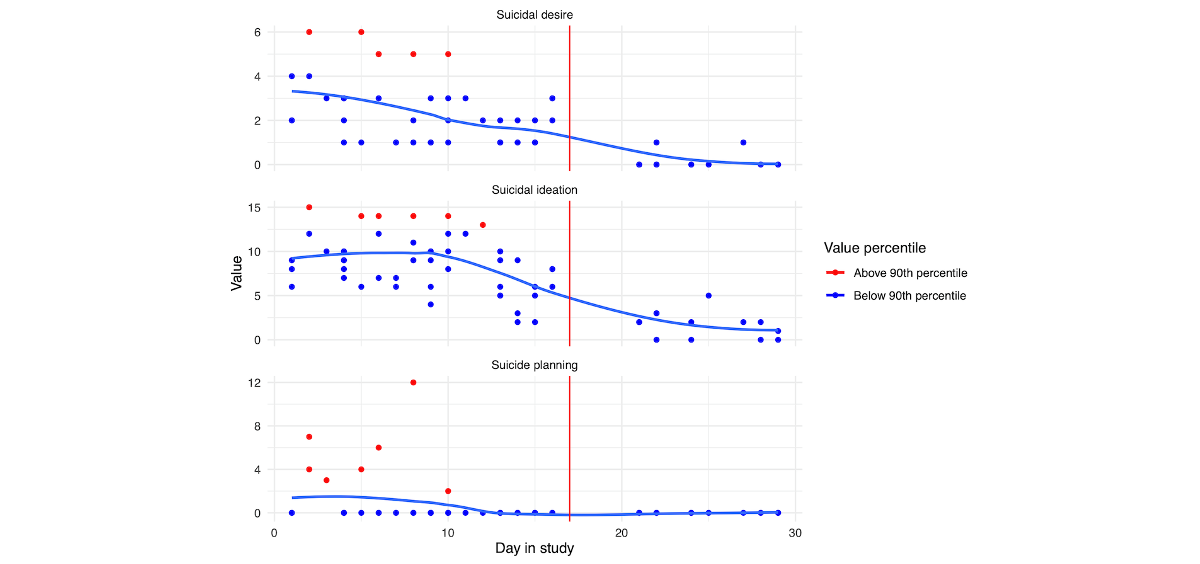
Person B: suicide ecological momentary assessment responses across days in the study. The blue line is a loess curve, while the vertical red line denotes the first day of hospitalization.

EMA responses related to suicidal ideation, planning, and desire for persons A and B are depicted in Figures 13 and 14, respectively; loess curves depict general trends, with red data points denoting higher relative values (ie, above the 90th percentile). For person A, we can see generally increasing trends for self-reported suicidal ideation and suicidal desire leading up to their hospitalization, with the highest reported values of suicidal ideation on the day of hospitalization. For person B, we can see elevated responses of self-reported suicidal ideation and planning leading up to their hospitalization. However, these elevations dissipate in the days immediately prior. As noted in Figures 2 and 3, we see lower response rates for surveys, including questions assessing suicidal ideation, planning, and desires in the days prior to hospitalization.

#### Passive Assessment

##### Risk Factor–Related Content

To assess passive assessment indicators of sleep, we attempted to link sleep and wake times, obtained via active assessment once per day, to screenshot collection timestamps. However, person A reported sleep and wake times on 16 days, while person B reported on 14 days. Given the small number of responses for each person (ie, approximately 50% missing data) and variability in their reported sleep and wake times, we did not feel confident calculating whether screenshots were collected during reported sleep times. Thus, we opted against reporting and visualizing this.

**Figure 15:**
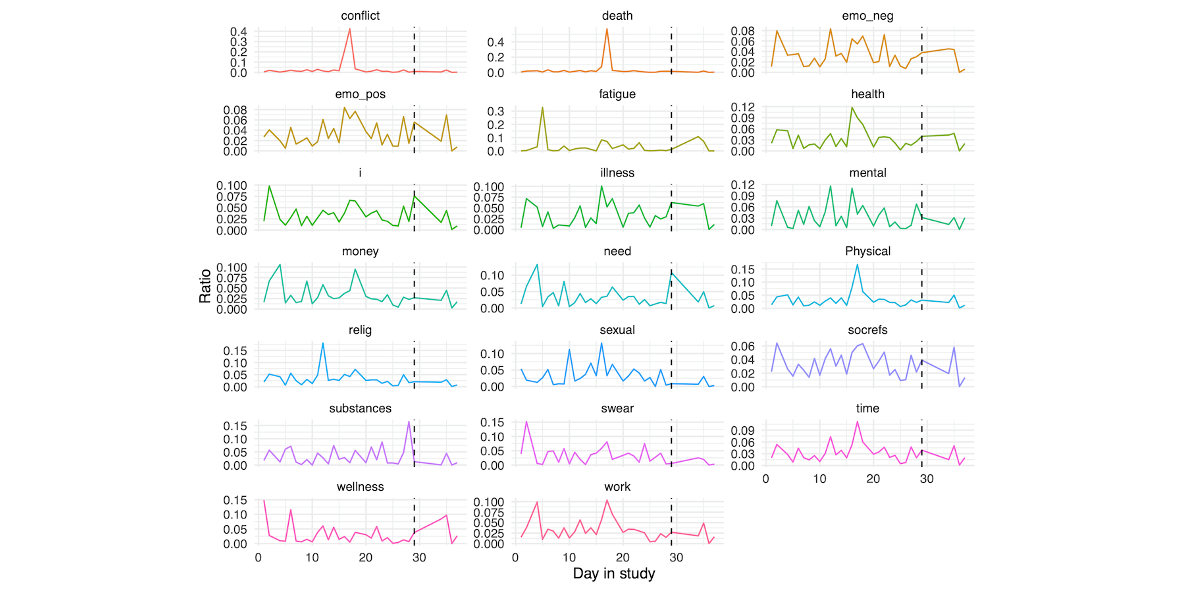
Person A: Linguistic inquiry and word count scores across days in the study. Note that scores are displayed as a ratio, using the daily score divided by the sum of all time points. The vertical black line denotes the first day of hospitalization.

**Figure 16:**
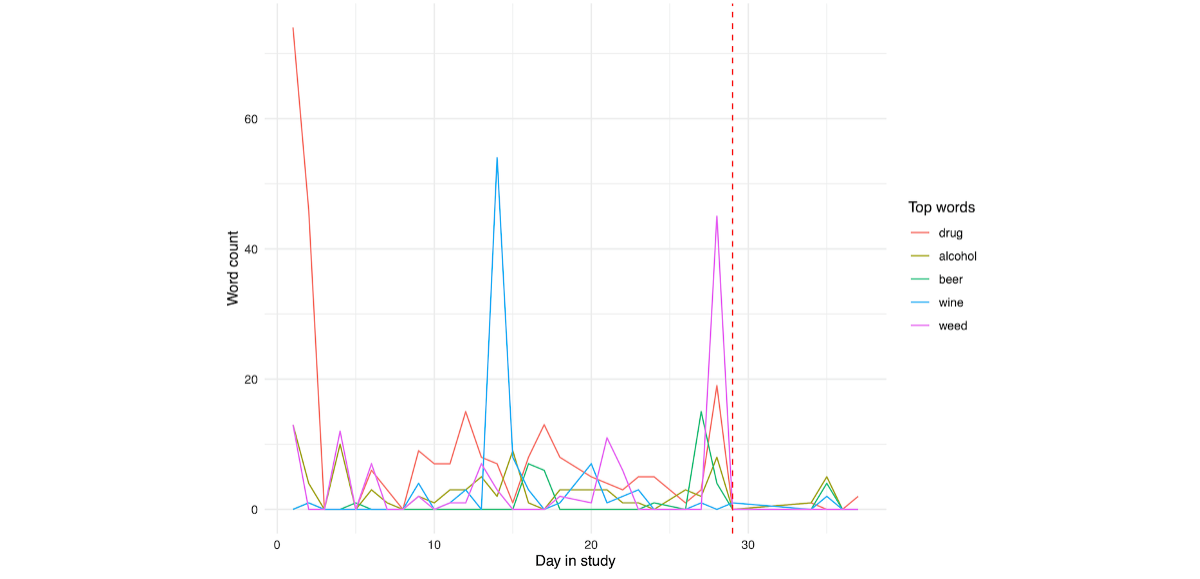
Person A: substance use scores across days in the study. The vertical red line denotes the first day of hospitalization.

For person A (Figure 15), we see peaks in several components leading up to their hospitalization, potentially most evident for the death and conflict scores. Probing further into the screenshot content during this day, we found that person A spent most of the day watching the TV show with “murder” in the title, likely contributing to these peaks. We also see a clear peak in the substances score prior to hospitalization. We created a substance-specific dictionary to further examine this (Multimedia Appendix 3). In applying this dictionary, we identified the top 5 words, and plot their use across the time period in Figure 16. To contextualize these findings, we examined a random selection of 1000 screenshots from the day prior to hospitalization. From these screenshots, it is evident that person A was experiencing a high degree of pain; this was corroborated by active responses of self-reported daily physical pain: although there were only 15 responses, their mean response was 74.4 (SD 16.2). They also disclosed through a SMS text message that an over-the-counter painkiller was not enough, and they could not get into a doctor to receive a prescription until the following month. Further, given the high identification of “weed” in screenshots the day prior to hospitalization, we extracted co-occurring text (ie, text from screenshots where “weed” was identified), and it became evident that person A was viewing posts about the medicinal use of marijuana on Facebook.

**Figure 17:**
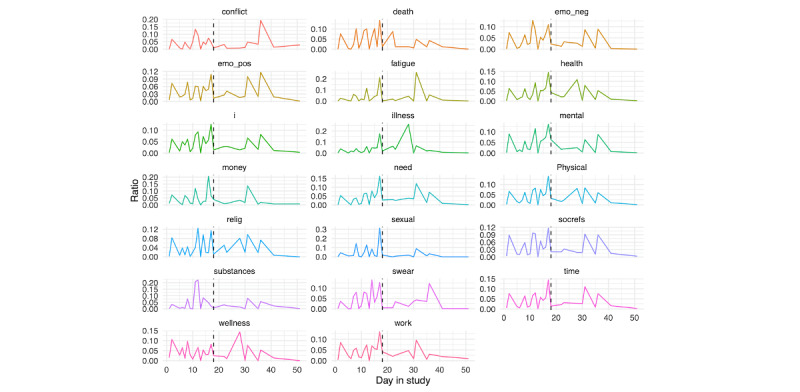
Person B: linguistic inquiry and word count scores across days in the study. Note that scores are displayed as a ratio, using the daily score divided by the sum of all time points. The vertical black line denotes the first day of hospitalization.

For person B (Figure 17), we see a number of components peak in the day prior to hospitalization. To contextualize these findings, we examined a random selection of 1000 screenshots from the day prior to hospitalization. We saw that they spent a large amount of time on a dating app (increase in sexual score in Figure 17) and conversing with individuals from that app. Further, like person A, person B was struggling with physical pain (specifically, head pain) and spent considerable time looking at health care providers and trying to identify a diagnosis, thus explaining the spike in a number of LIWC categories (ie, physical and health). Notably, they only completed 10 active responses of self-reported daily physical pain, making it hard to derive any conclusions from their average score of 26.8 (SD 23.5).

**Figure 18:**
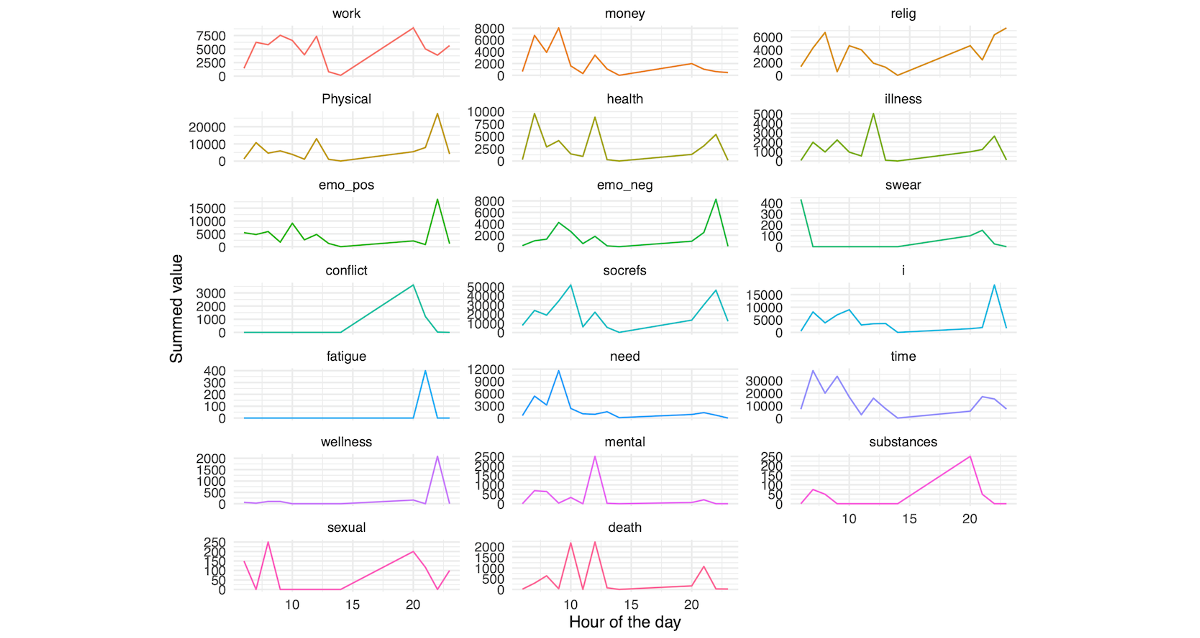
Person A: Linguistic inquiry and word count scores on the day before hospitalization.

We further examined risk factor text content on the day prior to hospitalization, as presented in Figures 18 (person A) and 19 (person B). For person A, a number of spikes in several risk factors were seen during the evening, whereas for person B, similar spikes were seen earlier in the day.

**Figure 19:**
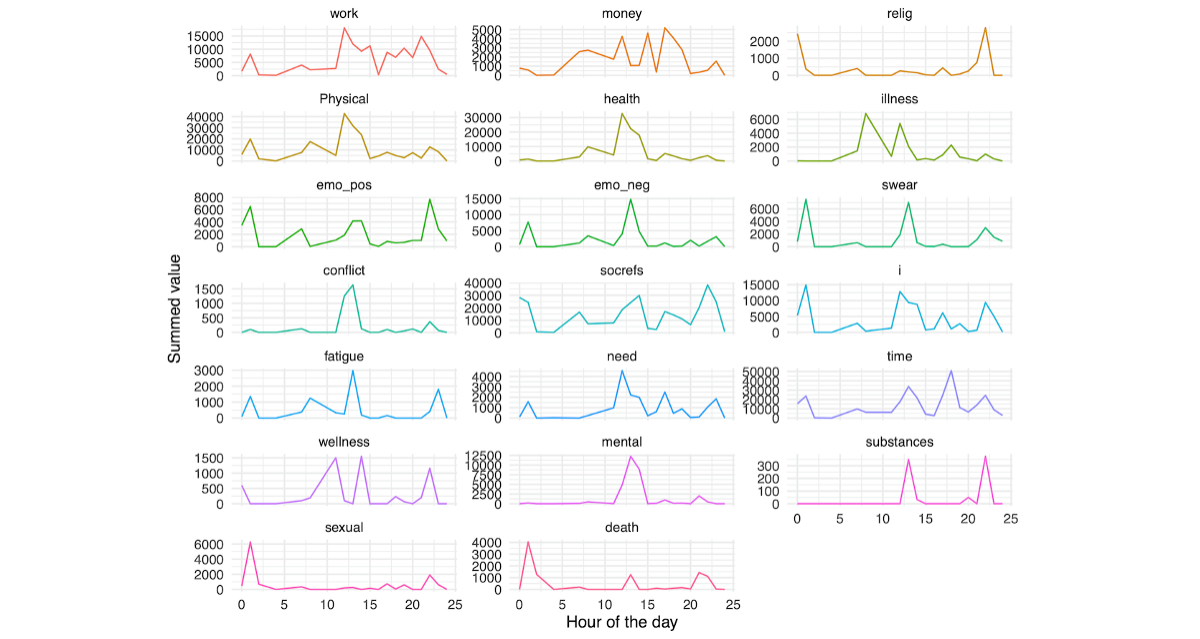
Person B: Linguistic inquiry and word count scores on the day before hospitalization.

##### Social Content

**Figure 20:**
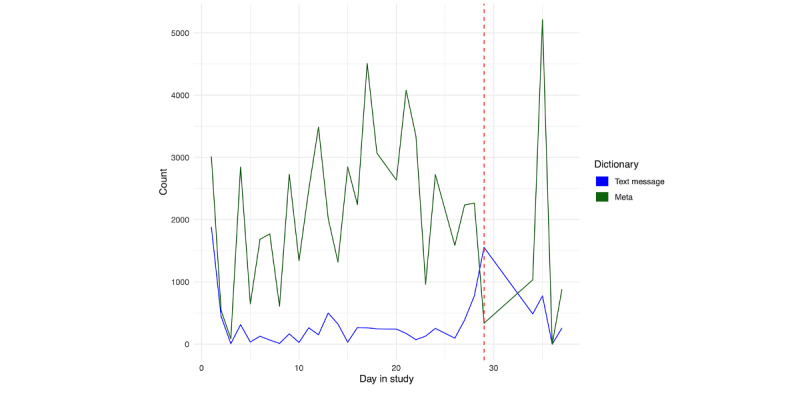
Person A: SMS text message and Meta usage across days in the study. The vertical red line denotes the first day of hospitalization.

**Figure 21:**
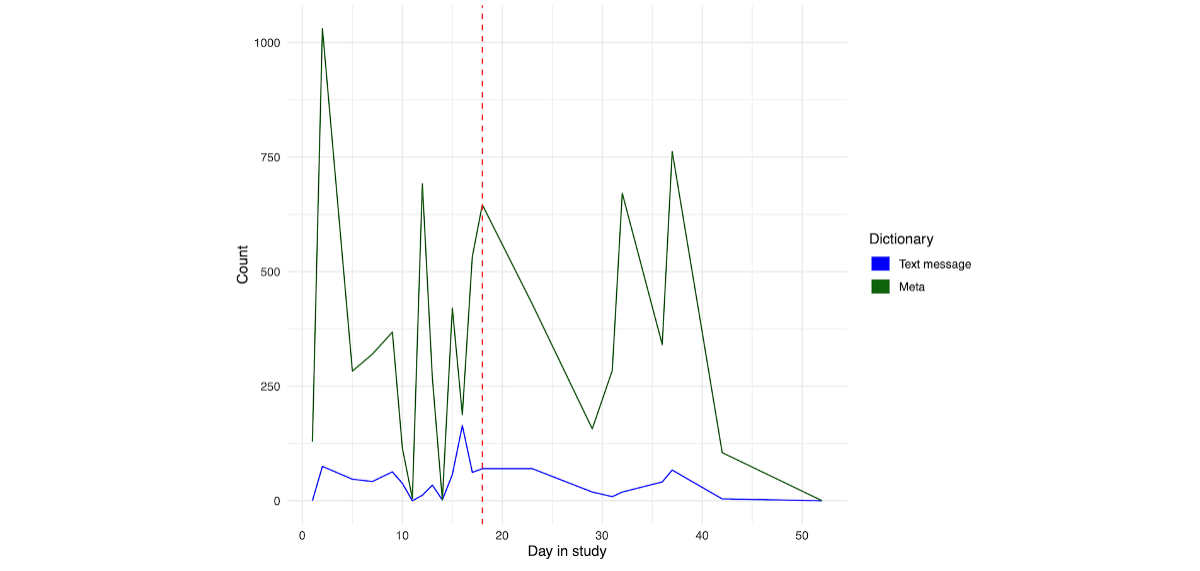
Person B: SMS text message and Meta usage across days in the study. The vertical red line denotes the first day of hospitalization.

Given the high amount of social contact for each person, we further examined changes in social behavior prior to hospitalization. Figure 20 shows the SMS text message and Meta (ie, Facebook, Instagram, and WhatsApp) use for person A. While the use of Meta apps does not show a clear change in the pattern of use relative to hospitalization, the use of text messaging peaks on the day of hospitalization. For person B (Figure 21), we see an increased use of Meta apps immediately prior to hospitalization, along with a slight increase in SMS text messaging in the days prior.

**Figure 22:**
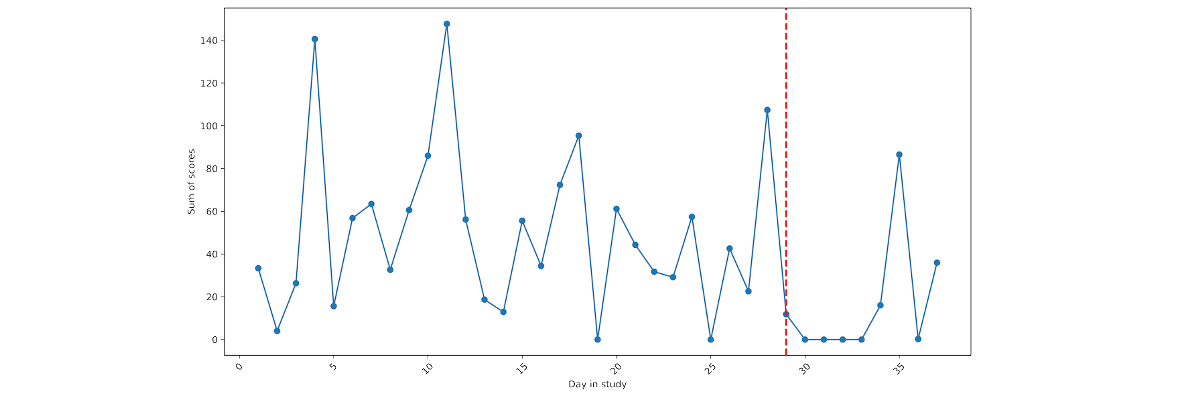
Person A: social content scores across day in the study. The vertical red line denotes the first day of hospitalization.

A similar conclusion can be drawn from the modeling of whether a screenshot contains social content in Figures 22 (person A) and 23 (person B). In examining a random sample of 1000 screenshots from the day of hospitalization, it appeared that person A was preparing for hospitalization by contacting friends to watch her dog and find a ride. Further, through disclosing her need for hospitalization, she was in frequent SMS text message conversations in which multiple individuals provided support for her decision. Alternatively, for person B, we see an increase in the use of social apps leading up to the day of hospitalization. However, on the day right before hospitalization, we do not see a large predicted total score of a number of screenshots containing social content.

**Figure 23:**
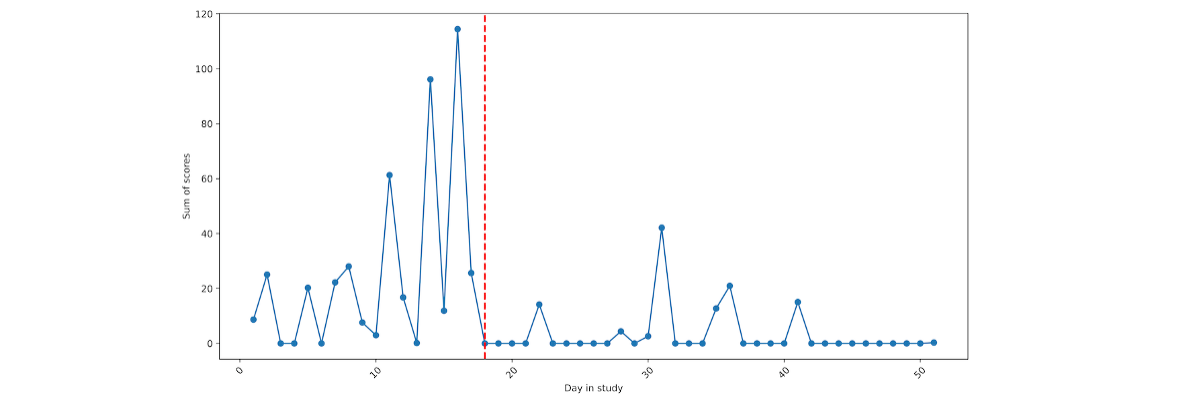
Person B: social content scores across day in the study. The vertical red line denotes the first day of hospitalization.

##### Suicide Language

**Figure 24:**
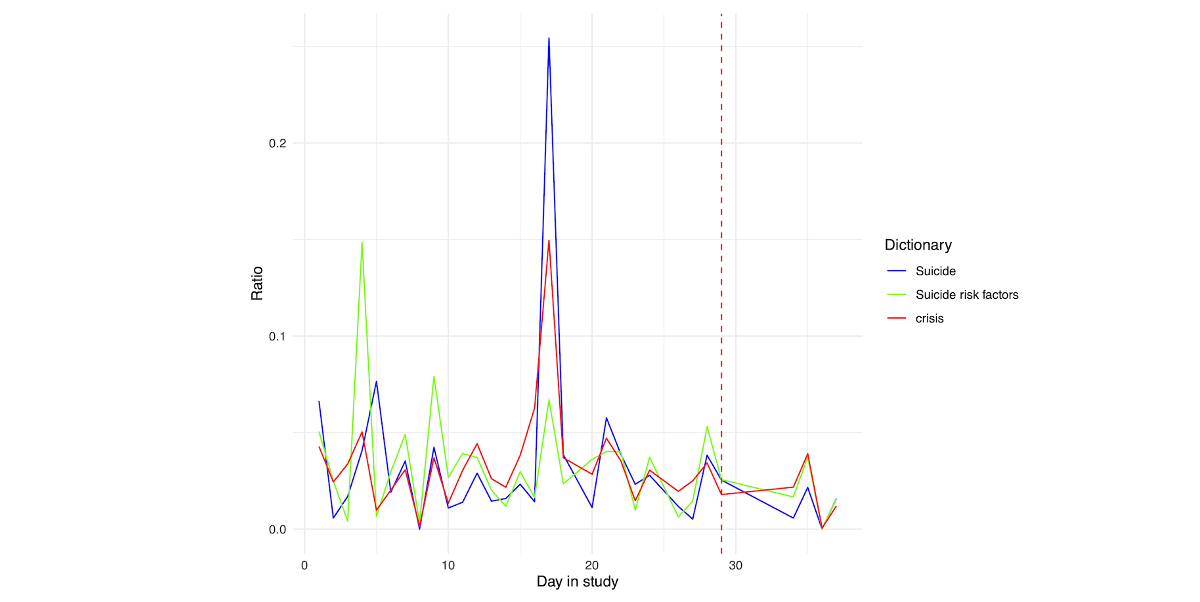
Person A: suicide risk scores across days in the study. Each score was derived from applying each dictionary in Multimedia Appendix 3 to text extracted from the screenshots. The vertical red line denotes the first day of hospitalization.

**Figure 25:**
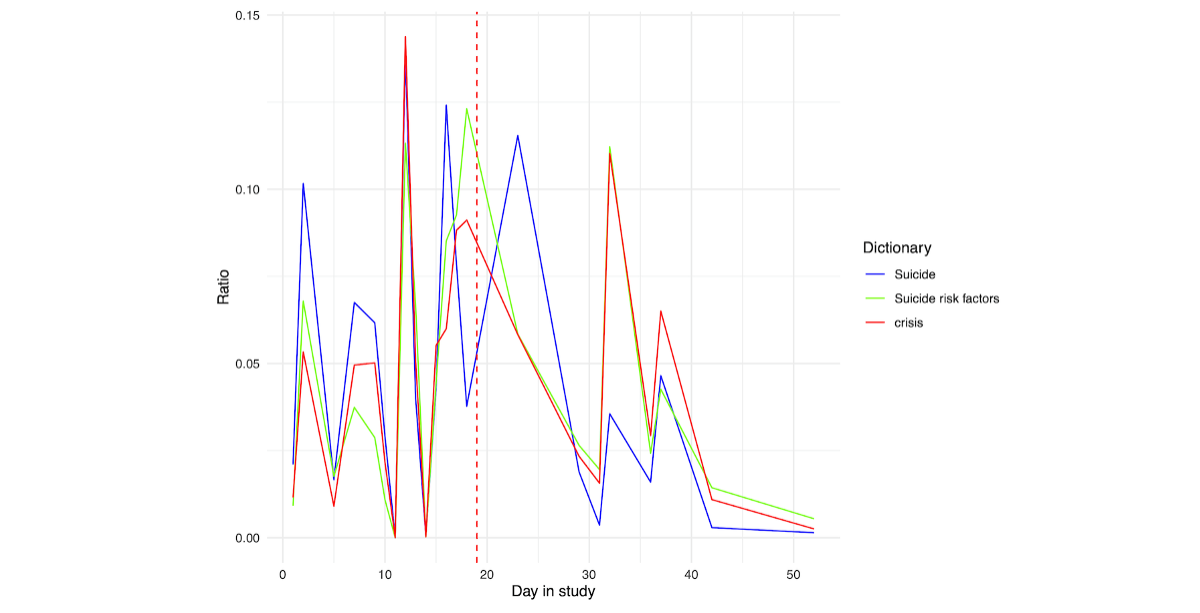
Person B: suicide risk scores across days in the study. Each score was derived from applying each dictionary in Multimedia Appendix 3 to text extracted from the screenshots. The vertical red line denotes the first day of hospitalization.

In applying the suicide-specific dictionaries, for person A (Figure 24), we only see the clear spike related to viewing the TV show. This is unsurprising given the other information gleaned from this participant’s screenshots, as their hospitalization was often discussed relative to pain, rather than suicidal thoughts or behaviors. Similarly, for person B (Figure 25), we see more of a peak with respect to risk factors and crisis words as opposed to suicide-specific words.

**Figure 26:**
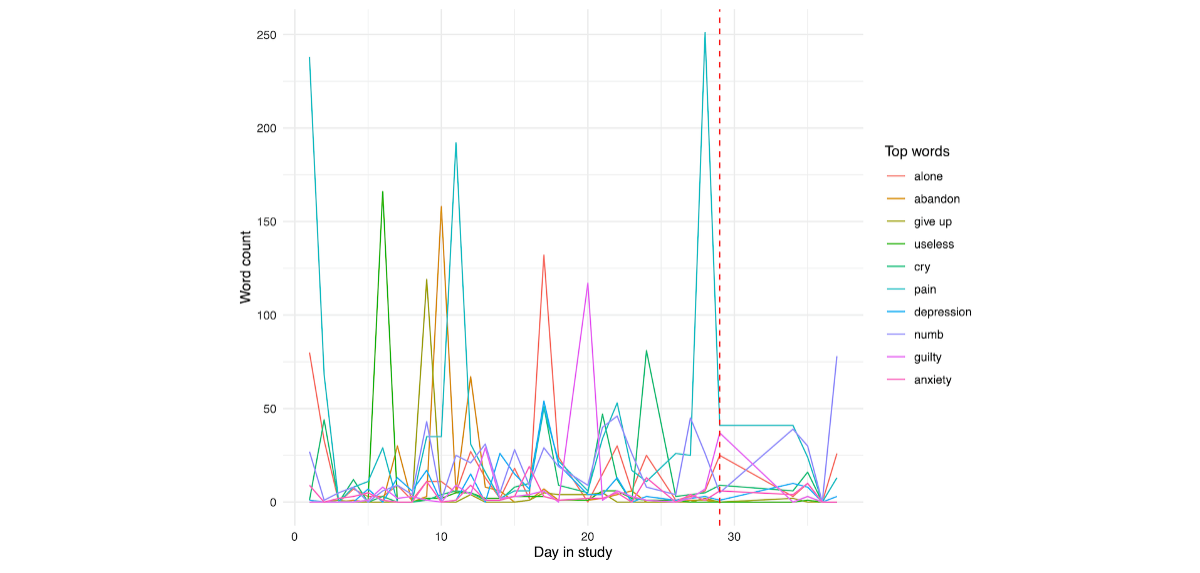
Person A: individual risk words across day in the study. The vertical red line denotes the first day of hospitalization.

**Figure 27:**
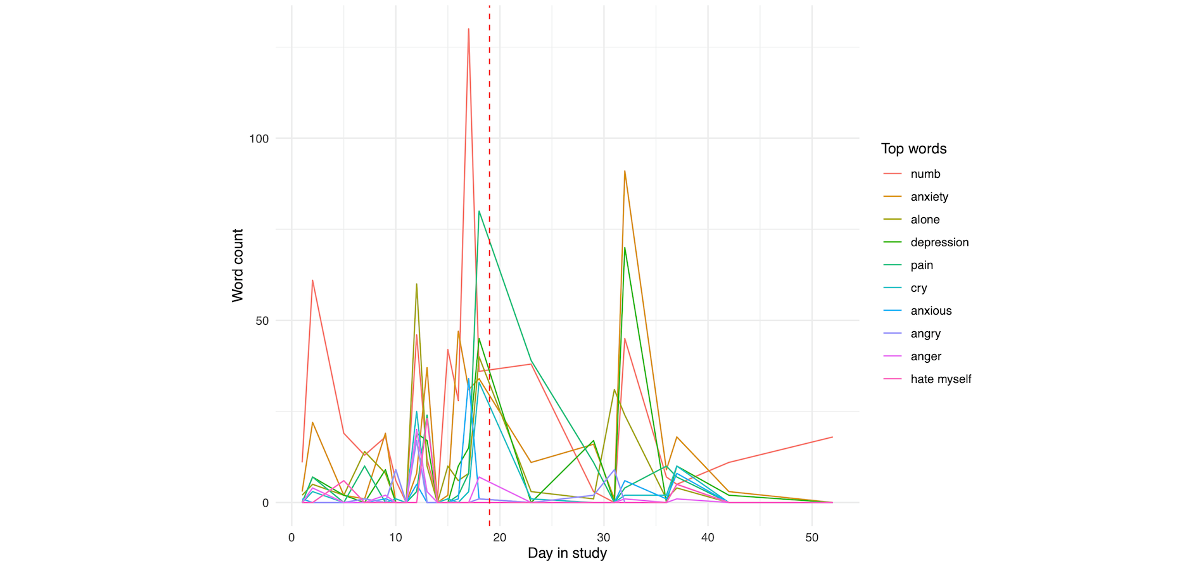
Person B: individual risk words across day in the study. The vertical red line denotes the first day of hospitalization.

**Figure 28:**
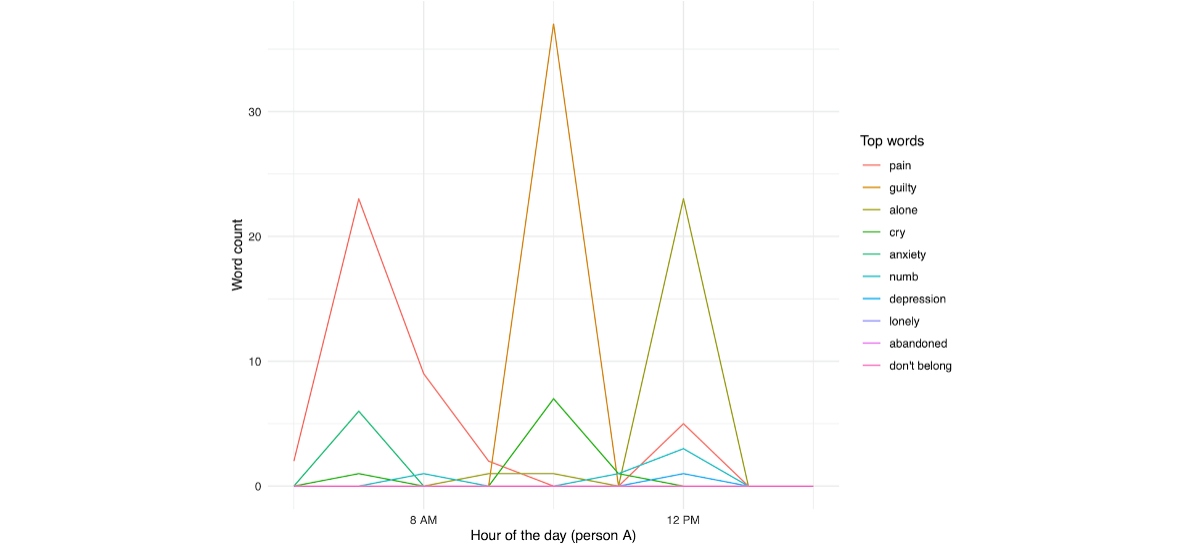
Person A: individual risk words in the day prior to hospitalization.

We then examined the top 10 endorsed individual risk words in Figures 26 and 27. For person A (Figure 26), we unsurprisingly see a large spike in pain on the day prior to hospitalization. This is further seen in counting the word identification within the day prior to hospitalization (Figure 28).

**Figure 29:**
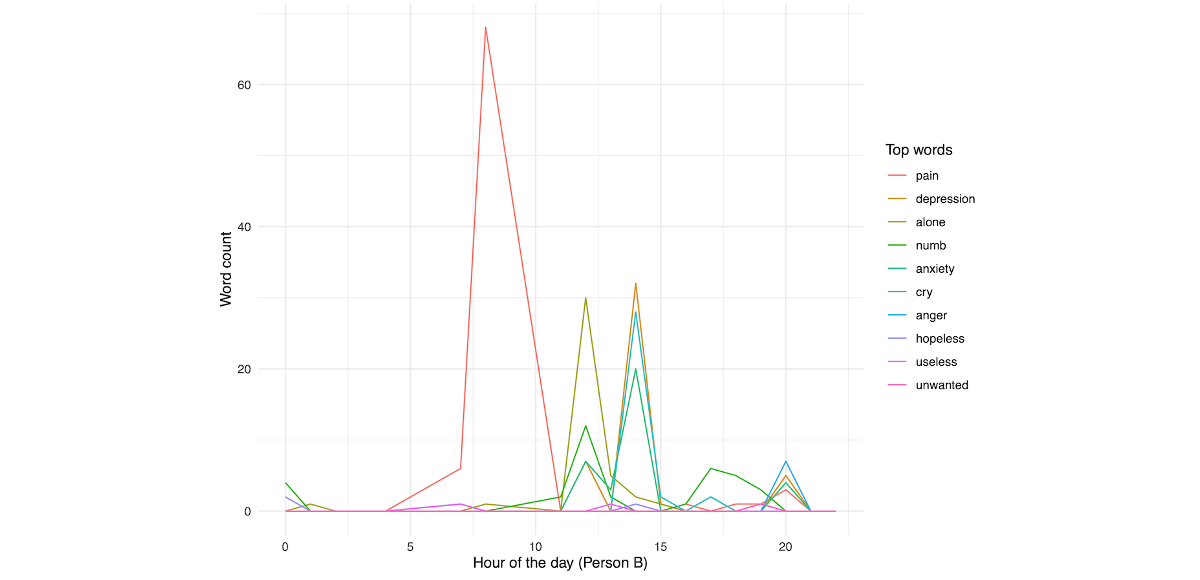
Person B: individual risk words in the day prior to hospitalization.

A peak in pain is followed by similar peaks in guilty and alone later in the day. Similar to person A, for person B (Figure 27), we see pain as peaking on the day before hospitalization, in addition to smaller, but noticeable peaks in anxiety, alone, and depression, as well as numb, in the few days prior. When examining the day prior to hospitalization (Figure 29), we see that the identification of pain primarily occurred earlier in the day, followed by smaller peaks in alone, depression, numb, and anxiety.

## Discussion

The goal of this comparative case review was to detail risk profiles obtained from both actively and passively collected smartphone data in the days leading up to a suicidal crisis. Through this, we also aimed to introduce the novel application of screenomics in suicide research. Our preliminary findings underscore the potential of passively collected data in understanding and predicting suicidal crises. The vast number of screenshots from each participant offers a granular look into their daily digital interactions, which, when combined with EMA assessments, provides a more comprehensive view of an individual’s psychological processes in the time leading up to a suicidal crisis. While this variegated picture of each individual’s life, as captured through a phone, makes a clean summarization impossible, it is worth noting some of the common elements evidenced in the data.

The first was decreased EMA compliance leading up to one’s hospitalization, muddying our understanding of an individual’s risk state, including the presence of STB. Perhaps most notably, this highlights the potential downfalls of relying solely on one’s ability and willingness to disclose their experiences in times of distress or crisis, having important implications for the prediction of, and intervention during, such risk states. We recognize this is conjecture based on the analysis of only 2 individuals and requires future research as, to our knowledge, this has not been previously assessed or tested in intensive longitudinal studies of STBs. However, if true, it has important consequences for the rising use of just-in-time interventions targeting suicide risk [34]. For example, relying on active (ie, EMA self-reported responses) may offer sufficient temporal granularity for intervening upon lower level risk states but may not be adequate for detecting crises given the burdensome design, particularly when in crises. Rather, as demonstrated in our case examples, personalized phone use patterns might be a more reliable indicator of impending suicidal crisis than EMA-assessed suicidal ideation.

A second takeaway from our analysis is that screenomics captured the salient precipitating factors for each suicidal crisis, which were not detected via EMA, due to a variety of reasons (ie, nonresponse and ceiling effects). The first illustrative example is that of physical pain. Self-reports via EMA were able to capture the role of physical pain in suicide risk, to some extent, for person A, who responded to approximately 54% of daily questions about pain. However, for both person A and B, texts extracted from their screenshots were able to more fully portray the significant, and dynamic, nature of this experience leading up to one’s hospitalization. While we recognize this risk process may be specific to the individuals selected for inclusion in this report, an effect with similar implications was demonstrated for the theoretically relevant risk factor of thwarted belongingness. For example, EMA responses with regard to thwarted belongingness were elevated across the majority of the study period, preventing an observation of increases, or spikes, prior to hospitalization. Alternatively, for both person A and person B, we see that alone, a central aspect to the construct of thwarted belongingness (ie, “I am alone” [26]), represented one of the top 10 risk factors words used and also demonstrated a spike in use during the day immediately preceding hospitalization. Together, these examples underscore the potential of passive data collection, such as screenomics, to tap into risk processes that are not evident from, or demonstrated by, self-report assessments. Indeed, the low-burden nature of such passive assessment may allow for a more nuanced detection of a wide array of potential risk factors, lending to meaningful personalized risk detection.

Finally, and possibly the most surprising element was a general increase in phone use leading up to the hospitalization, including the increase in social use. It remains unclear as to whether this was a causal factor—that is, contributed to suicide risk—or was the result of seeking social support for their distress, making arrangements to be hospitalized (pet care or work), or other possible help-seeking behaviors, to cope with their escalating suicide risk. Further, the function of social phone use at or near times of suicidal crises may be person or situation specific (ie, idiographic). For example, person A appeared to be engaging in help-seeking behavior via SMS text message (eg, arranging rides) prior to hospitalization, while person B largely engaged in social and dating app usage, potentially contributing to acute interpersonal stressors. It will be useful to further develop our data processing pipeline to further explore the relationships between phone use, particularly social use, and suicide risk.

Of note, we purposely did not report any results from statistical models. While it is becoming more commonplace to apply linear models at the individual level [35], we opted against this. Our main focus for this comparative case report was on identifying risk patterns preceding hospitalization; the forgoing statistical models allowed us to explore a greater number of risk pathways in a more in-depth manner. Further, each participant had a limited number of assessments, with an increasing number of missed prompts leading up to the hospitalization (Figures 2 and 3), making the application of linear models difficult. Moreover, applying a longitudinal model to components derived from screenshots presents a number of challenges due to the high dimensionality, and uncertainty regarding forms of validity (eg, in naming components).

Within suicide research, and particularly intensive longitudinal research, ethical consideration of participant safety is a primary concern [36]. The incorporation of active assessments has the clear benefits of face validity to specific suicide questions (ie, “Are you planning to attempt suicide today?”), along with straightforward processing to derive risk score cutoffs that indicate required follow-up by study staff and the ability to readily access such data (ie, manually examining responses). As noted above, there are potential issues with relying on active assessment responses (ie, noncompliance [37,38]). However, the use of passively collected data (broadly defined) also presents a number of challenges for use in ascertaining risk. The first is the clear lack of published research on which data sources are valid markers of risk. Second, while some data points are easily calculated (ie, the number of screenshots and steps walked), others would require complex and expensive processing pipelines to process data in real time. For instance, in screenshots presented in this case study, this would require automatic decryption of uploaded images to a server that is integrated with a graphics processing unit (or central processing unit) architecture that can apply pretrained models to generate new data sources (eg, SMS text messages) or predictions (eg, suicide risk scores based on the text). Similarly, most smartwatches require manual syncing with software before data are uploaded to the cloud, thus precluding real-time analysis. Thus, a large degree of additional research is required before incorporating various types of passive assessment into real-time participant safety protocols.

A number of limitations to this paper should be mentioned. First, as this is the first comprehensive investigation of smartphone screenshot data to improve our understanding of suicide risk, aspects of our approach are preliminary in nature, including the use of specific (ie, suicide, risk factor, and substance) dictionaries. It will be important for further validation of these dictionaries prior to wide-scale applications. Our focus on studying 2 individuals with a complex case profile limits the generalizability of our findings across the wider spectrum of severity of suicidal ideation, planning, and behaviors. Consequently, our results may not fully represent the experiences of those at lower levels of suicide risk (ie, not experiencing a suicidal crisis). The findings may also not generalize to individuals at a similar risk level but do not consider psychiatric hospitalization as a treatment option. Further research with larger and more diverse samples is necessary to validate and extend our findings to a broader population. Finally, we only included one form of passive sensing, screenomics, while a number of other modalities, such as geo-location or watch-based assessments (ie, sleep, steps, and heart rate), have seen wide application in clinical research [39].

In conclusion, the primary aim of this paper was to provide an in-depth examination of both passive and active data modalities from 2 individuals who were hospitalized for suicidal crises. We hope this paper has introduced the value of collecting passive data, specifically screenshots, at an intensive timescale, as well as highlighting the potential of its ability to incrementally add to our understanding of suicide risk timescale and underlying processes.

## Appendix

Multimedia Appendix 1 Ecological Momentary Assessment (EMA) questions to assess risk factors.

Multimedia Appendix 2 Linguistic Inquiry and Word Count (LIWC) components [30].

Multimedia Appendix 3 Words used in specific dictionaries.

## Abbreviations

EMA: ecological momentary assessment
HIPAA: Health Insurance Portability and Accountability Act of 1996
LIWC: linguistic inquiry and word count
STB: suicidal thoughts and behavior

## Data Availability

The screenshot data set collected and analyzed during this study is not available from the authors at this time. However, it will eventually be accessible through the NIMH National Data Archive. Derivative data sets used to create each figure are available from the corresponding author on reasonable request.
