# Supplementary material for: Examining Passively Collected Smartphone-Based Data in the Days Prior to Psychiatric Hospitalization for a Suicidal Crisis: Comparative Case Analysis
Source: JMIR Form Res. 2024 Mar 20;8:e55999. doi: 10.2196/55999 (PMC10993130; doi:10.2196/55999)
Supplement: Multimedia Appendix 1 [file formative_v8i1e55999_app1.docx]

### Multimedia Appendix 1. Ecological Momentary Assessment (ECA) questions to assess risk factors.

### Momentary emotional state

Each EMA prompt assessed momentary positive and negative affect via the Positive and Negative Affect Schedule [27], which includes ten items assessing positive affect states and ten items assessing negative affect states on a 5-point Likert scale.

### Momentary theoretically-relevant risk factors.

Each EMA prompt assessed the momentary theoretically--relevant affect states of: “useless,” “like a burden for others,” “like I do not belong,” and “lonely”[28]. Each item was answered on a 5-point scale. One item also assessed the presence of an interpersonal conflict or interpersonally stressful situation since the last prompt.

### Momentary empirically-relevant risk factors

Each EMA prompt assessed current urges to drink alcohol, use drugs, and harm oneself, or self-injury, without the intent to die. Each item was answered on a 5-point Likert scale.

### Daily empirically-relevant risk factors

The first EMA prompt of the day assessed sleep quality via four questions: sleep time, wake time, presence of nightmares, and subjective quality of sleep on a 5-point Likert scale. The last prompt of each day assessed the presence of alcohol use and self-injury, as well as the level of physical pain experienced across the day (answered on a 100-point VAS scale).
