# Supplementary material for: Examining Passively Collected Smartphone-Based Data in the Days Prior to Psychiatric Hospitalization for a Suicidal Crisis: Comparative Case Analysis
Source: JMIR Form Res. 2024 Mar 20;8:e55999. doi: 10.2196/55999 (PMC10993130; doi:10.2196/55999)
Supplement: Multimedia Appendix 2 [file formative_v8i1e55999_app2.docx]

Multimedia Appendix 2. Linguistic Inquiry and Word Count (LIWC) components [31].

| Category | Description from Manual and/or exemplars |
| --- | --- |
| work | work, school, working, class |
| money | business*, pay*, price*, market* |
| religion | god, hell, christmas*, church |
| physical | medic*, food*, patients, eye* |
| health | medic*, patients, physician*, health |
| illness | hospital*, cancer*, sick, pain |
| wellness | healthy, gym*, supported, diet |
| mental health (mental) | mental health, depressed, suicid*, trauma* |
| substances | beer*, wine, drunk, cigar* |
| sexual | sex, gay, pregnan*, dick |
| death | death*, dead, die, kill |
| Positive Emotion (emo_pos) | good, love, happy, hope |
| Negative Emotion (emo_neg) | bad, hate, hurt, tired |
| swear | shit, fuckin*, fuck, damn |
| Interpersonal Conflict (conflict) | fight, kill, killed, attack |
| Social References (socrefs) | you, we, he, she |
| 1st person singular (i) | I, me, my, myself |
| fatigue | tired, bored, don’t care, boring |
| need | have to, need, had to, must |
| time | when, now, then, day |
