# Supplementary material for: Examining Passively Collected Smartphone-Based Data in the Days Prior to Psychiatric Hospitalization for a Suicidal Crisis: Comparative Case Analysis
Source: JMIR Form Res. 2024 Mar 20;8:e55999. doi: 10.2196/55999 (PMC10993130; doi:10.2196/55999)
Supplement: Multimedia Appendix 3 [file formative_v8i1e55999_app3.docx]

Multimedia Appendix 3. Words used in specific dictionaries.

| Suicide | Risk Factor | Substance Use |
| --- | --- | --- |
| Kill myself | alone | Drug |
| Kill yourself | lonely | Alcohol |
| Want to die | abandoned | Marijuana |
| Wanna die | Don’t belong | Cocaine |
| die | Dont belong | Heroin |
| suicide | unwanted | Meth |
| suicidal | hopeless | Weed |
| Suicide note | Give up | Beer |
| Don’t want to live | Hate myself | Wine |
| Don’t wanna live | useless | Vodka |
| Dont wanna live | burden | Cannabis |
| End it all | cry | Opium |
| death | crying | Lsd |
|  | pain | Ecstasy |
|  | depressed | Amphetamine |
|  | depression | benzodiazepine |
|  | numb |  |
|  | trapped |  |
|  | angry |  |
|  | agitated |  |
|  | restless |  |
|  | guilt |  |
|  | guilty |  |
|  | shame |  |
|  | anxiety |  |
|  | anxious |  |
